# High-sensitivity electrochemical immunosensor for anti-SARS-CoV-2 IgG detection using screen-printed carbon/cerium oxide-gold electrode

**DOI:** 10.5599/admet.3182

**Published:** 2026-04-10

**Authors:** Melania Janisha Devi, Ratu Shifa Syafira, Shabarni Gaffar, Irkham Irkham, Yasuaki Einaga, Yeni Wahyuni Hartati

**Affiliations:** 1 Department of Chemistry, Faculty of Mathematics and Natural Sciences, Padjadjaran University, Jl. Raya Bandung-Sumedang Km 21, Jatinangor, Sumedang, West Java 45363, Indonesia; 2 Department of Chemistry, Keio University, 3-14-1 Hiyoshi, Yokohama, 223-8522, Japan

**Keywords:** Electrochemical immunoassay, ceria-gold nanohybrid, modified carbon electrode, SARS-CoV-2 IgG antibodies

## Abstract

**Background and purpose:**

Serological assays are essential for evaluating immune responses, including donor screening, vaccine efficacy, and antibody persistence. However, conventional methods are time-consuming and require centralized laboratories. This study aimed to develop a sensitive and rapid electrochemical immunosensor based on a cerium oxide-gold nanocomposite (CeO_2_-Au) modified screen-printed carbon electrode (SPCE) for the detection of anti-SARS-CoV-2 IgG, while elucidating the underlying electrochemical sensing mechanism.

**Experimental approach:**

CeO_2_-Au nanocomposites were synthesized and characterized using UV-Vis, SEM, TEM-EDX, and FTIR. The immunosensor was fabricated by immobilizing SARS-CoV-2 Spike receptor-binding domain (RBD) onto the modified SPCE. Electrochemical responses were evaluated using differential pulse voltammetry (DPV) and electrochemical impedance spectroscopy (EIS) with K_3_[Fe(CN)_6_] as a redox probe.

**Key results:**

The CeO_2_-Au nanocomposite enhanced electron transfer and provided a high surface area for biomolecule immobilization. The sensing mechanism is governed by modulation of interfacial electron transfer: binding of IgG to immobilized RBD forms an insulating immunocomplex layer, increasing charge transfer resistance and suppressing faradaic current. The sensor exhibited a wide linear range of 0.01 to 10^3^ ng mL^-1^, a low detection limit of 2.475 pg mL^-1^, good stability, and reliable 97.3 to 108.56 % recovery in serum samples.

**Conclusion:**

This immunosensor proposed as a sensitive and reliable platform for IgG detection. The study advances understanding of signal transduction mechanisms in nanocomposite-based immunosensors and highlights their potential for rapid serological diagnostics. However, broader clinical validation and selectivity against complex interferents remain necessary.

## Introduction

In recent years, the COVID-19 (Coronavirus Disease 19) pandemic, caused by the severe acute respiratory syndrome coronavirus 2 (SARS-CoV-2), has had a profound global impact [1]. SARS-CoV-2 continues to circulate worldwide, and long-term monitoring strategies remain necessary. Vaccination programs have played a central role in mitigating disease severity and transmission. Since vaccination stimulates the production of virus-specific antibodies, evaluation of post-vaccination immune responses remains an important analytical objective. The kinetics and durability of antibody titers following vaccination have been widely reviewed [2], and key questions persist regarding antibody persistence, comparative vaccine performance, and optimal revaccination timing.

Serological testing has therefore become an essential tool for evaluating vaccine-induced immune responses [3]. Serological assays quantify antibodies in blood samples [4] and are widely used to assess the production of SARS-CoV-2-specific antibodies after infection or vaccination [5]. In addition to monitoring vaccine efficacy and durability, serological testing supports identification of reactive donors for convalescent plasma therapy and contributes to broader public health strategies [6].

Studies have shown that serum IgG concentrations vary widely across individuals, vaccination status, and time post-vaccination. Before vaccination, the IgG concentrations were generally very low or undetectable. After vaccination, IgG levels typically range from 1 to 1000 μg mL^-1^, depending on the vaccine type and the individual's immune response [2,7]. This range served as the target concentration for the serological analysis in this study.

The gold-standard method for serological testing is the plaque-reduction neutralization test (PRNT) [8]. However, PRNT results take 2-4 days, require biosafety level 3, and must be performed by experienced laboratory personnel [9]. Other methods include chemiluminescent immunoassay (CLIA) [10], enzyme-linked immunosorbent assay (ELISA) [5,11,12], chemiluminescent microparticle immunoassay (CMIA) [9,13,14] and lateral flow assay (LFA) [15] have been done on previous research. However, the LFA results are qualitative. CMIA and CLIA must be performed by trained personnel and require several hours to complete. Therefore, fast, sensitive, and accurate serological methods that can be used in public places or at home, such as biosensors, are needed [16]. Biosensors are advantageous because of their superior detection and sensitivity limits compared to ELISA [17,18]. Electrochemical biosensors based on screen-printed carbon electrodes (SPCE) have attracted considerable attention due to their ability to operate in complex matrices with high accuracy, specificity, and sensitivity [19,20]. In addition, SPCE can be easily modified with nanomaterials to improve the electroanalytical performance and biosensor sensitivity [21,22].

Cerium oxide (CeO_2_) exhibits good biocompatibility [23,24]. Biosensors require biocompatible materials to maintain their bioreceptor activity [25]. In addition, CeO_2_ exists in two oxidation states, Ce^3+/4+^, thus giving it protein adsorption properties based on the charge on its surface [26]. In recent years, functionalized CeO_2_ amine groups (NH_2_) have attracted attention because they can facilitate the formation of nanocomposites, in which NH2 sites on the surface of CeO_2_ can bind to metals [27,28]. Nanocomposites are the combination of two or more materials to form new materials with better properties. Gold nanoparticles (AuNPs) are widely used to improve the electrical conductivity of materials [29]. In this study, SPCE was modified with CeO_2_-Au nanocomposites to enable protein adsorption for immobilization and to improve signal response. The use of CeO_2_-Au nanocomposites for detecting IgG Anti-SARS-CoV-2 has not been previously reported. Moreover, the synthesis of CeO_2_-Au nanocomposites was adapted from existing methods, yielding a material with enhanced sensitivity and specificity for IgG detection.

The accuracy and reliability of serological methods depend largely on the choice of targeted SARS-CoV-2 antigen and testing format [30]. Biosensors based on antigen-antibody interactions are called immunosensors [31]. The main target of antibodies against coronavirus antigens is the spike protein, of which the receptor-binding domain (RBD) is the main epitope for the immune response. Therefore, the Spike RBD protein is used as a bioreceptor in immunosensors. Antibody tests can be performed against SARS-CoV-2 IgG, IgM and IgA [30,32]. However, IgG binds to antigens with greater affinity and specificity than other antibodies [33]. IgG accounts for approximately 75 % of serum antibodies and has the longest serum half-life, making it more suitable for serosurveillance studies [34-36]. The production of antibodies follows a specific timeline. IgM is typically the first antibody produced during an immune response, followed by IgG, which appears later but persists longer in the serum. IgA, on the other hand, plays a critical role in mucosal immunity but is less stable in the systemic circulation. Studies have shown that anti-S and anti-RBD IgG levels are significantly higher than those of IgM or IgA in vaccinated patients [37], which aligns with their role in long-term immunity. This characteristic makes IgG a preferred target for serological diagnostics and vaccine efficacy assessment.

Research on electrochemical immunosensors for the detection of SARS-CoV-2 antibodies and other biomolecules has advanced considerably in terms of sensitivity, stability, and applicability to real samples. Chen *et al.* [29] developed a GCE/Ag@CeO_2_-Au immunosensor for carcinoembryonic antigen detection, demonstrating good stability over four weeks under low-temperature storage. Rahmati *et al*. [38] reported an SPCE/Ni(OH)_2_-based immunosensor capable of detecting SARS-CoV-2 antibodies in serum with recovery values of 99 to 103 %, highlighting the feasibility of nanomaterial-modified SPCE platforms for serological analysis. De Brito *et al.* [39] systematically investigated the influence of temperature, incubation time, and stabilizers on the stability of immunosensors for Salmonella detection, emphasizing the critical role of controlled storage parameters. Meanwhile, Sari *et al*. [40] developed an SPCE-Au-based aptasensor for SARS-CoV-2 RBD detection in saliva and demonstrated its performance across different sample matrices. In addition, Zakiyyah *et al*. [41] demonstrated that AuNP-modified SPCE prepared by drop-casting and spray-coating methods significantly improves electron transfer and sensitivity for SARS-CoV-2 biosensing applications . Similarly, Swara *et al.* [42] reported a CeO_2_ nanoparticle-based electrochemical immunosensor on SPCE for biomolecular detection, highlighting the potential of CeO_2_ nanostructures to enhance sensor performance through improved surface reactivity and electron transfer properties.

Although these studies illustrate the versatility of Au- and oxide-modified SPCE systems, challenges remain regarding long-term stability, controlled surface chemistry, and consistent signal amplification in antibody detection. In particular, most previously reported systems rely on single-component nanomaterials or focus primarily on analytical sensitivity without systematic evaluation of storage parameters and shelf life.

In this work, we present a rationally designed SPCE/CeO_2_-Au nanocomposite immunosensor for the electrochemical detection of anti-SARS-CoV-2 IgG. The CeO_2_-Au hybrid nanostructure was selected to combine the high conductivity and catalytic properties of Au nanoparticles with the oxygen vacancy-rich surface and chemical stability of CeO_2_, thereby promoting enhanced charge transfer, signal amplification, and improved interfacial stability. The SARS-CoV-2 Spike RBD protein was immobilized onto the SPCE/CeO_2_-Au surface *via* cysteamine, exploiting electrostatic interactions between surface NH_2_ groups and negatively charged amino acid residues and the C-terminus of the RBD. This configuration enabled specific IgG recognition, and antibody binding was quantified electrochemically using differential pulse voltammetry (DPV) and electrochemical impedance spectroscopy (EIS) based on modulation of electron transfer in the K_3_[Fe(CN)_6_] redox system.

Compared with previously reported Au-modified or oxide-modified SPCE immunosensors [38,43], the present platform emphasizes not only analytical sensitivity and dynamic range but also structural stability and storage robustness. The combination of CeO_2_ and Au provides a distinct nanostructure composition that supports efficient electron transfer while maintaining surface integrity, contributing to reproducible performance over time and offering a simplified architecture compatible with decentralized serological testing.

Given that biomolecular stability is influenced by abiotic factors such as temperature, pressure, and pH, as well as biotic factors such as enzymatic degradation [44], storage conditions are critical for the reliability of immunosensors. As demonstrated by De Brito *et al.* [39], stabilizer composition and environmental parameters significantly affect device performance. Therefore, beyond analytical characterization, we systematically investigated the influence of stabilizer solution, temperature, packaging, and humidity on immunosensor stability. A fractional factorial design was employed to evaluate the contribution of these four factors. The shelf life of the device was then determined under selected storage conditions. This approach extends previous stability-focused studies [45,46] by integrating material design with structured storage optimization.

Finally, the developed immunosensor was evaluated using human serum samples obtained after SARS-CoV-2 vaccination to assess recovery and method validity, compared with a reference CMIA assay. These results further support the feasibility of the proposed CeO_2_-Au SPCE platform as a stable and sensitive proof-of-concept system for serological antibody detection. The immunosensor test results were compared with those obtained using the methods used at the Prodia Clinical Laboratory, Bandung, Indonesia.

## Experimental

### Material

The materials used were demineralized water, chloroauric acid (HAuCl_4_.3H_2_O) (synthesized by the Chemical Analysis and Separation Laboratory December 28, 2018, Indonesia [41]), ammonium hydroxide (NH_4_OH) (Merck), bovine serum albumin (BSA) (Sigma Aldrich), cerium oxide (CeO_2_) (Sigma Aldrich), cysteamine (Merck), ethanol (C_2_H_5_OH) (Merck), human serum sample, monoclonal antibody (anti-SARS-CoV-2 m-IgG) (My BioSource), phosphate buffer saline (PBS) pH 7,4 (Merck), potassium ferricyanide (K_3_[Fe(CN)_6_]) (Sigma Aldrich), (3-aminopropyl)triethoxysilane (APTES) 98 % (Sigma Aldrich), potassium chloride (KCl) (Merck), SARS-CoV-2 Spike RBD (GenScript, Singapore), sodium azide (NaN_3_) (Merck), sodium ethylenediaminetetraacetic (Na_2_EDTA) (Merck), trisodium citrate dihydrate (Na_3_C_6_H_5_O_7_) (Merck), and toluene (C_7_H_8_) (Merck).

### Instrumentation

The apparatus used were FT-IR spectrometer (Perkin Elmer Spectrum 100), hot plate (IKA C-MAG HS 7), magnetic stirrer (Eppendorf), mini spin (Eppendorf), weighing balance (Mettler Toledo AL204), centrifuge (Corning), sonicator (Ultrasonic Cleaner), Zimmer Peacock potentiostat using PSTrace 5.9 software, UV-VIS spectrophotometer (Thermo scientific), SPCE (GSI Technologies, USA) with a carbon working electrode (diameter 5 mm), a carbon auxiliary electrode, and an Ag/AgCl reference electrode, SEM (Hitachi TM3000), SEM-EDX (Inspect S50, FEI), TEM-EDX (Tecnai T12, FEI), XRD (D8 Advance, Bruker), and glassware.

### Procedure

#### Synthesis of gold nanoparticles

AuNPs were synthesized following the Turkevich method [40,47,48]. Briefly, 134.3 μL of 55.85 mM chloroauric acid (HAuCl_4_·3H_2_O) was diluted to 10 mL with deionized water in an Erlenmeyer flask. The solution was heated to boiling under continuous stirring (600 rpm). Upon reaching the boiling point, 1.7 mL of 1 % (w/v) sodium citrate was rapidly added. The mixture was maintained at boiling for 10 min under stirring until the solution colour changed to burgundy, indicating the formation of colloidal AuNPs. The colloidal AuNPs were cooled to room temperature (RT) and stored in amber glass bottles at 4-5 °C until further use. The AuNPs were characterized using UV-Vis spectrophotometry.

#### Functionalization of cerium oxide with (3-aminopropyl)triethoxysilane

CeO_2_ powder (1.0 g) was dispersed in 25 mL of ethanol and sonicated for 15 min. Subsequently, 15 mL of concentrated ammonia solution (25 %) was added rapidly under stirring. The mixture was stirred for 6 h at room temperature (25 °C). The resulting CeO_2_-OH precipitate was collected by centrifugation at 8000 rpm (≈7500*g*) for 10 min. The solid was washed three times with 20 mL of deionized water and three times with 20 mL of ethanol. The washed material was dried at 50 °C for 12 h. For silanization, 0.2 g of CeO_2_-OH was dispersed in 15 mL of toluene, followed by the addition of 50 μL APTES. The mixture was sonicated for 30 min and then refluxed at 60 °C under stirring (500 rpm) for 5 h. The product was separated by centrifugation at 8000 rpm (≈7500*g*) for 10 min and washed three times with 20 mL ethanol and twice with 20 mL deionized water. The resulting CeO_2_-NH_2_ was dried at 50 °C for 12 h and characterized using FTIR spectroscopy.

#### Synthesis of cerium oxide-gold nanocomposite

CeO_2_-NH_2_ (10 mg) was dispersed in 25 mL colloidal AuNP solution and sonicated for 30 min. The suspension was stirred at room temperature (25 °C) for 12 h to allow electrostatic interaction between AuNPs and amine-functionalized CeO_2_. The nanocomposite was collected by centrifugation at 9000 rpm (≈8500 × g) for 15 min, then washed three times with 20 mL of deionized water and twice with 20 mL of ethanol. The CeO_2_-Au precipitate was dried at 50 °C for 12 h and stored in a desiccator at room temperature prior to use. The nanocomposite was characterized using FTIR, SEM-EDX, TEM-EDX, and UV-Vis spectrophotometry.

#### Screen-printed carbon electrodes modification with CeO_2_-Au

The SPCE surface was rinsed with deionized water and dried under nitrogen flow. Surface activation was performed using UV irradiation (254 nm) for 15 min. A 40 μL suspension of CeO_2_-Au (2 mg/mL in deionized water) was drop-cast onto the working electrode and dried at room temperature for 60 min. The modified electrode (SPCE/CeO_2_-Au) was rinsed gently with 1 mL deionized water and air-dried for 30 min. Electrochemical characterization was performed using 40 μL of 10 mM K_3_[Fe(CN)_6_] in 0.1 M KCl. SEM-EDX was used to examine surface morphology.

#### Optimization of parameters affecting experiments

The factors selected for optimization in this experiment were the incubation concentration of SARS-CoV-2 RBD (X1), incubation time of SARS-CoV-2 RBD (X2), and incubation time of SARS-CoV-2 IgG (X3). Each factor was designed with three levels: the lowest level (-1), the middle (0), and the highest (+1). These factors are listed in Table 1. The response to the suggested experiment was then processed, and the optimal value for each factor was determined using the Box-Behnken experimental design in Minitab 19.

**Table 1.** Optimization of factors influencing experimental conditions using Box-Behnken

Factor	Unit	Level		
		-1	0	+1
RBD concentration, μg mL^-1^		1.25	3.25	5.25
RBD incubation time, min		2	36	70
IgG incubation time, min		2	9	16

### Immunosensor assembly

**Figure 1.**
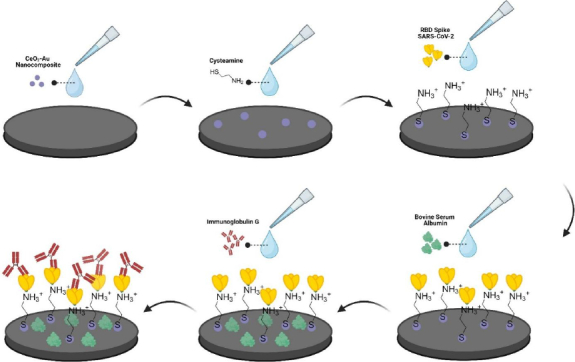
Illustration of immunosensors for anti-SARS-CoV-2 IgG developed in this study

SPCE/CeO_2_-Au was modified with 40 μL of 0.1 M cysteamine and incubated for 2 h at room temperature in the dark. The electrode was rinsed with 1 mL of deionized water and dried for 30 min. RBD solution (20 μL, 3.25 ng μL^-1^) was immobilized and incubated at room temperature for 43 min. The electrode was washed twice with 1 mL PBS (pH 7.4). Blocking was performed using 20 μL of 1 % (w/v) BSA for 15 min. Excess BSA was removed by washing twice with PBS (1 mL each) (Figure 1). Each assembly step was monitored by DPV and EIS using 10 mM K_3_[Fe(CN)_6_] in 0.1 M KCl. Modified electrodes were stored at 4-5 °C in sealed containers with silica gel until use (no longer than 24 h before measurement unless otherwise specified).

### Determination of analytical parameters

The Anti-SARS-CoV-2 IgG at varying concentrations (0, 0.01, 0.1, 1, 10, 100 and 1000 ng mL^-1^) was tested on the immunosensors. Twenty microliters of anti-SARS-CoV-2 IgG solution was dripped onto the electrode and incubated for 11 min at room temperature. Residual IgG that did not interact with the RBD was rinsed with PBS (pH 7.4).

The resulting electrochemical response was measured by DPV and EIS using a redox system of a potassium ferricyanide solution (K_3_[Fe(CN)_6_], 10 mM in KCl, 0.1 M) [43]. Next, a calibration curve was constructed between concentration and the difference in the average current peak (Δ*I*) for each measurement, yielding the equation *y* = b*x* + a.

The detection limit (LoD) and quantification limit (LoQ) were calculated by calculating the standard deviation of the intersection of the regression y-line (*σ*) using Equations (1) and (2):



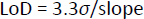

(1)




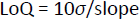

(2)


where the slope is obtained from the regression equation *y* = b*x* + a

Precision and accuracy were determined by measuring 1 ng mL^-1^ of IgG six times. From the measurement results, the average difference in peak current (Δ*I*) and the measurement standard deviation were obtained. Precision is expressed as the relative standard deviation (RSD, %), while Accuracy, % is expressed as relative error (Error, %) following Equations (3) to (5) [49,50]:



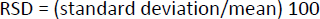

(3)




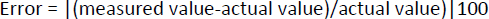

(4)




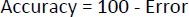

(5)


### Immunosensor selectivity test

IgG and H5N1 antigens were prepared at 1, 10, 100 and 1000 ng mL^-1^. A 20 μL aliquot was applied and incubated for 11 min. The electrode was washed twice with PBS (1 mL each) before DPV measurement.

### Immunosensor stability test

Stability was evaluated under different storage conditions (Table 2). Stabilizer solution (20 μL; 0.2 % sodium azide (w/v), 1 mM Na_2_EDTA, 1 mM BSA in PBS) was applied where indicated. Electrodes were stored at 4 to 5 °C or room temperature under controlled humidity conditions. Weekly measurements were performed for six weeks using 1 ng mL^-1^ IgG.

**Table 2.** Immunosensor storage was performed for 7 days for the fractional factorial design.

Storage factors	Low level (-1)	High level (+1)
Stabilizing solution	Not	Yes
Housekeeping	Open (exposed to particles)	Closed (without particle display)
Temperature	4 to 5 °C	RT
Moisture	Low (controlled with silica gel)	Usual

The current response of the immunosensor after storage was compared with that of the standard/non-storage immunosensor (0 days). Storage conditions that resulted in lower current changes were selected and immunosensor shelf-life studies were conducted.

Immunosensors were stored for six weeks, with a measurement period of seven days. The immunosensors were then tested against 20 μL of anti-SARS-CoV-2 IgG at a concentration of 1 ng mL^-1^. The electrochemical response was measured by DPV using a redox system of potassium ferricyanide solution (K_3_[Fe(CN)_6_] 10 mM in KCl 0.1 M) [47]. Then, the stability of the immunosensor (Stability, %) was calculated using Equation 5 [38,39,51,52]



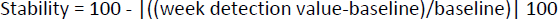

(5)


### Human serum sample analysis

The prepared serum samples were analysed using the standard addition method. Ten microliters of the sample were diluted to 2.5 mL, then 10 μL was diluted again to 2.63 mL. A diluted sample (50 μL) was added to each tube containing standard IgG at various concentrations. Each tube was diluted to 500 μL. The variations in the standard concentrations after dilution were 0.01, 0.1, 1 and 10 ng mL^-1^. The immunosensor that had been blocked on the nonspecific side with bovine serum albumin (BSA) was rinsed with PBS (pH 7.4). Then, 20 μL of the sample solution was dropped onto the electrode and incubated at room temperature for 11 min. DPV was then measured using a redox system of potassium ferricyanide solution (K_3_[Fe(CN)_6_] 10 mM in 0.1 M KCl). The results of measuring IgG levels in serum samples were compared with those from the serological method used at the Prodia Clinical Laboratory.

After that, recovery (*R* / %) of each standard concentration in the serum sample was determined using equation 6 as follows [38]:



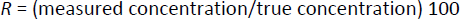

(6)


## Results and discussion

### UV-Vis spectrophotometry characterization

**Figure 2.**
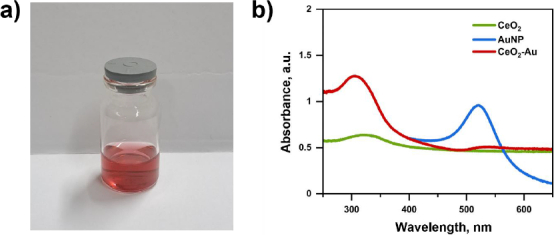
(a) Photograph of synthesized colloidal gold nanoparticles (AuNPs). (b) UV-Vis absorption spectra of CeO_2_, AuNPs, and CeO_2_-Au nanocomposites, showing the characteristic surface plasmon resonance peak of AuNPs at ~520 nm and the absorption features of CeO_2_ in the UV region

UV-Vis absorption spectroscopy is the most widely used method for characterizing the optical properties and electronic structure of materials. The absorption of ultraviolet and visible radiation was used to determine the electronic transition of the compound. The UV-Vis spectrum of commercial CeO_2_ showed an absorption peak at 308 nm, as shown in Figure 2b. Absorption in the UV region arises from the charge transition between O (2p) and Ce (4f) in CeO_2_. Based on the literature, the maximum uptake peak for CeO_2_ is generally between 290 and 360 nm [53].

AuNP formation can be analysed using UV-Vis spectrophotometry with a maximum absorption wavelength of approximately 400 to 600 nm [54]. The AuNP colloids synthesized in this study exhibited an absorption peak at a wavelength of 521 nm (Figure 2). Colloidal AuNPs have a characteristic burgundy colour (Figure 2a) [55]. At the nanometre scale, electron clouds can oscillate on the surfaces of particles and absorb electromagnetic radiation at specific energies. This resonance is known as surface plasmon resonance (SPR) or plasmon nanoparticle absorbance, and is a consequence of its small size [56].

UV-Vis absorption spectra of CeO_2_-Au nanocomposites showing conformity with commercial CeO_2_ and the synthesized AuNPs for comparison. It was observed that the CeO_2_-Au nanocomposites have two absorption peaks at 250 to 350 nm (*λ*_max_ = 308 nm) and 500 to 550 nm (*λ*_max_ = 540 nm). The UV-Vis absorption spectra of CeO_2_-Au nanocomposites show two characteristic absorption bands at 250-350 nm (*λ*_max_ = 308 nm), corresponding to CeO_2_, and 500-550 nm (*λ*_max_ = 540 nm), corresponding to the surface plasmon resonance (SPR) of AuNPs. Compared to the colloidal AuNPs (*λ*_max_ = 521 nm), a red shift to 540 nm was observed after composite formation. This shift may be attributed to changes in the local dielectric environment, interfacial interactions between AuNPs and CeO_2_, and possible particle aggregation effects, rather than solely to an increase in particle size.

### Scanning electron microscopy and transmission electron microscopy

**Figure 3.**
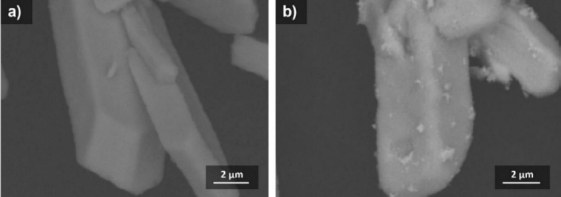
SEM micrographs of (a) pristine CeO_2_ and (b) CeO_2_-Au nanocomposites. The CeO_2_ structures exhibit rod-like microstructure morphology, while Au nanoparticles are uniformly distributed on the CeO_2_ surface

Scanning electron microscopy (SEM) characterization was performed to determine the morphology of the CeO_2_-Au nanocomposites. Figure 3(b) shows the results of the analysis of the CeO_2_-Au nanocomposites with a magnification of 10,000×, revealing morphological differences compared to pristine CeO_2_, indicating surface modification after AuNP incorporation. These results were compared with the SEM images of CeO_2_ shown in Figure 3(a). The surface of CeO_2_ after the composite contains small AuNP grains. These results are consistent with previously reported research [28].

**Figure 4.**
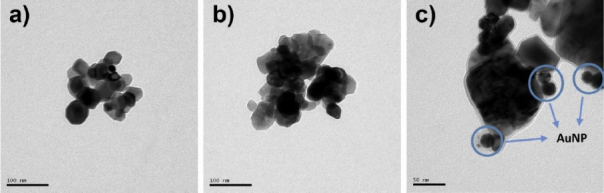
TEM micrographs of (a) pristine CeO_2_, (b) ultrasonically treated CeO_2_ and (c) CeO_2_-Au nanocomposites, showing improved particle dispersion after treatment and uniform deposition of Au nanoparticles on the CeO_2_ surface

The results of transmission electron microscopy (TEM) characterization are shown in Figure 4. CeO_2_, before being composited with AuNPs was done without and with grinding to see whether the effect of grinding can make the commercial CeO_2_ size smaller. With scouring, the surface and shape of CeO_2_ become less corrugated and coarser (Figure 4b) than before scouring (Figure 4a). CeO_2_ without scour was then selected and composited with AuNPs (Figure 4c). AuNPs produced by the Turkevich method are spherical. Based on representative TEM micrographs, the AuNPs have an average diameter of ~20 nm, with relatively narrow size variation.

**Figure 5.**
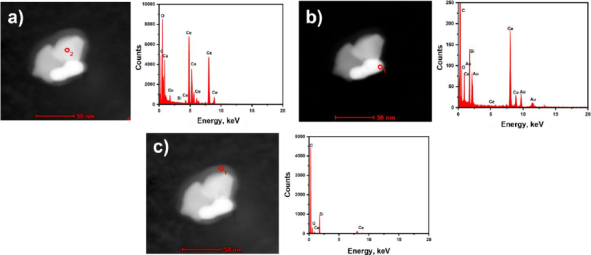
TEM images and EDX characterization of (a) CeO_2_, (b) AuNPs and (c) APTES-functionalized CeO_2_ layers. The CeO_2_ exhibits nanoscale crystalline domains, while AuNPs show spherical morphology with narrow size distribution. The APTES-modified CeO_2_ maintains structural integrity, and the appearance of Si and N peaks in the EDX spectra confirms successful surface functionalization

These results are supported by the TEM-EDX analyses presented in Figure 5. In Figure 5(a), the detected elements are predominantly Ce and O, confirming that the analysed region corresponds to CeO_2_. In Figure 5(b), the presence of Au signals indicates the localization of Au nanoparticles within the analysed area. In Figure 5(c), the detection of C and Si elements is attributed to the presence of APTES functional groups associated with the CeO_2_-Au nanocomposite surface.

Although quantitative particle size distribution analysis (*e.g.* histogram analysis or DLS measurement) was not performed, qualitative TEM observations and SPR characteristics support the formation of AuNP-decorated CeO_2_ nanocomposites. Future work will include comprehensive statistical particle size analysis to further correlate optical properties with nanoparticle dimensions.

### CeO_2_ functionalization with APTES

**Figure 6.**
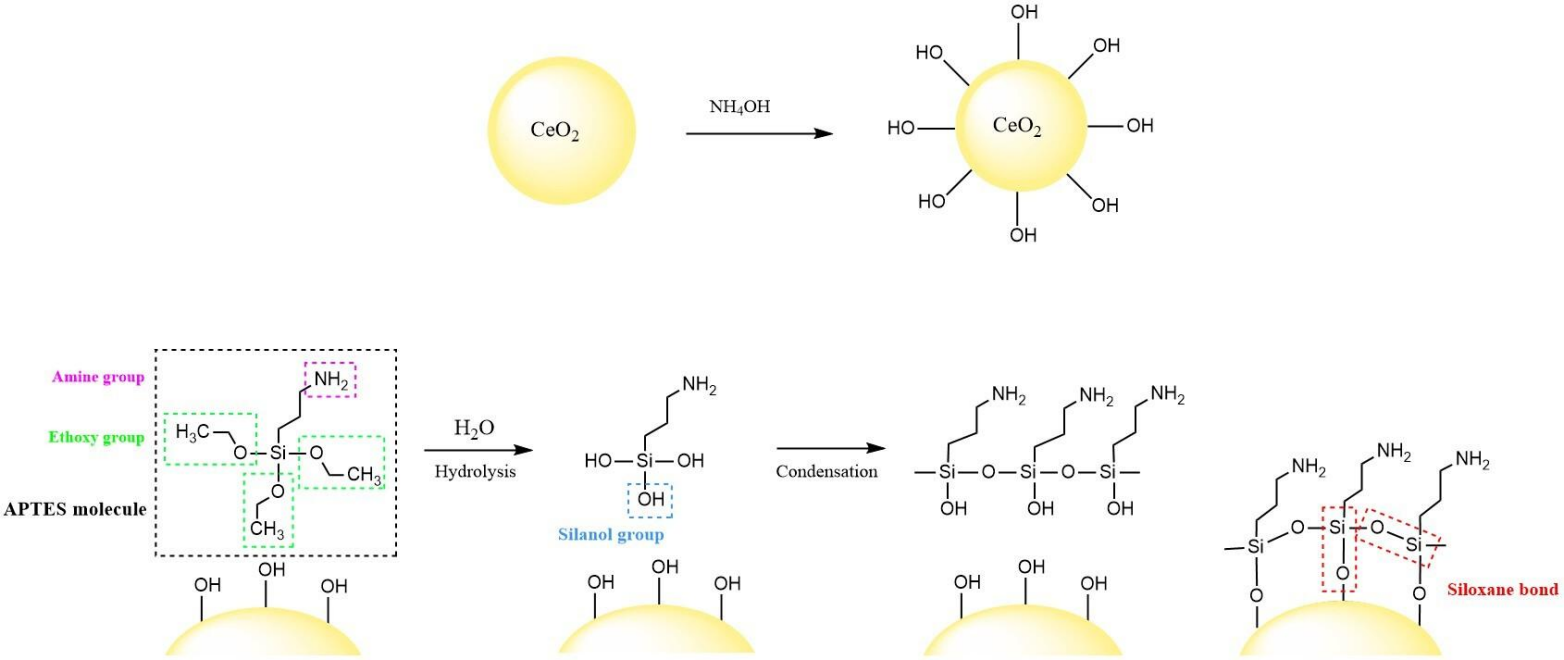
Proposed mechanism for APTES functionalization of CeO_2_, illustrating the hydrolysis of APTES, formation of silanol groups, and subsequent condensation with surface hydroxyl groups of CeO_2_ to form stable Si-O-Ce bonds

Functionalization of CeO_2_ with APTES facilitates the formation of nanocomposites with AuNPs [27]. Activation of the cerium surface with hydroxyl groups improves APTES attachment. The interaction between CeO_2_ and APTES involves three steps: (1) hydrolysis of the ethoxy group into a hydroxyl group, (2) condensation with the formation of siloxane bonds, (3) formation of hydrogen bonds with OH groups on the substrate and (4) formation of a covalent bond (Si-O-Ce) between the silicon in organosilane and CeO_2_ with the loss of water molecules [57]. The synthesis of CeO_2_-APTES is illustrated in Figure 6.

**Figure 7.**
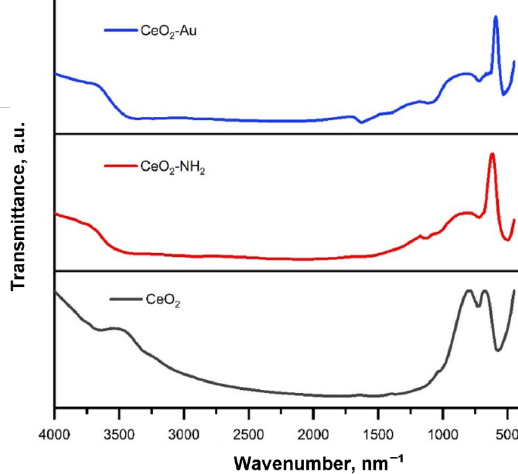
FTIR spectra of pristine CeO_2_, amine-functionalized CeO_2_ (CeO_2_-NH_2_) and CeO_2_-Au nanocomposites, showing characteristic Ce-O lattice vibrations in the low wavenumber region and the appearance of amine-related bands after surface functionalization. Changes in peak intensity and/or slight shifts after Au incorporation confirm successful surface modification and nanocomposite formation. FTIR characterization of CeO_2_, CeO_2_-NH_2_, and CeO_2_-Au nanocomposites

The APTES coating on CeO_2_ was characterized using IR spectroscopy to determine the functional groups present in the sample. The analysis was performed by comparing the spectra of CeO_2_, CeO_2_-NH_2_, and CeO_2_-Au nanocomposites. The resulting IR spectra are shown in Figure 7.

In the IR CeO_2_ spectrum, there is an absorption band at 563 cm^-1^, which originates from the Ce-O strain vibration and shifts to the 520 cm^-1^ area after APTES [58]. The absorption band at 3600 cm^-1^ in the CeO_2_ spectrum originates from vibrations of water molecules or surface -OH functional groups [53]. In the CeO_2_-NH_2_ and CeO_2_-Au spectra, absorption bands at wavenumbers of 1000 to 1200 cm^-1^ are observed, indicating the presence of Si-O strain vibrations. In addition, absorption bands at 1382, 1536, and 3560 cm^-1^ appeared, which were derived from the bending vibrations of C-H, N-H, and strained O-H, respectively [23,58]. From the spectrum analysis, it can be concluded that APTES was successfully functionalized on the CeO_2_ surface.

### Screen-printed carbon electrodes surface characterization

**Figure 8.**
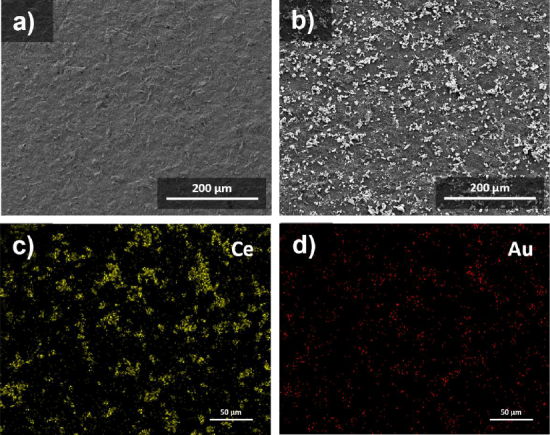
SEM characterization results from (a) SPCE bare, (b) SPCE/CeO_2_-Au and EDX mapping of SPCE/CeO_2_-Au for (c) Ce and (d) Au

SEM characterization was performed to determine the morphology of the SPCE before and after the modification. Figure 8 shows the SEM results for the SPCE/CeO_2_-Au surface compared to the bare SPCE. The surface of SPCE after modification is white, indicating a CeO_2_-Au nanocomposite (Figure 8b), compared to before modification (Figure 8a). This is confirmed by the results of EDX mapping for Ce and Au elements scattered on the surface of SPCE (Figure 8c), indicating that SPCE modification with CeO_2_ was successful.

**Figure 9.**
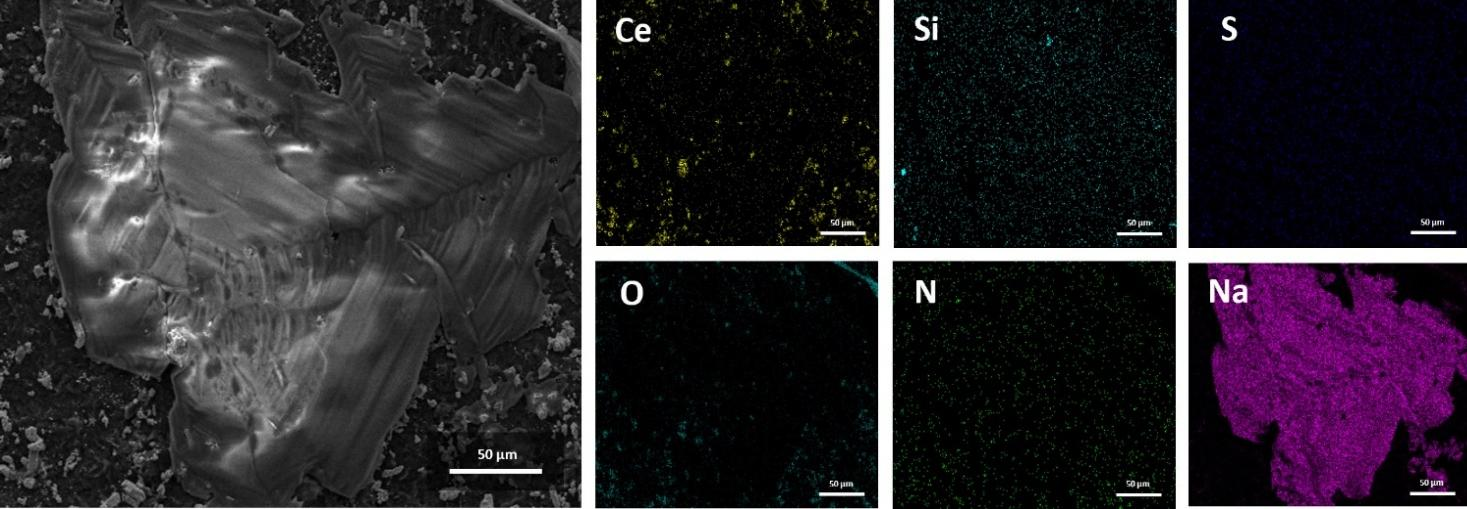
SEM-EDX mapping characterization results from SPCE/CeO_2_-Au/Cysteamine/SARS-CoV-2 Spike RBD. EDX elemental mapping showing the distribution of Ce, Si, S, O, and N, respectively, on the electrode surface. Ce and O originate from the CeO_2_-Au nanocomposite, while S and N indicate the presence of cysteamine and amino acid residues from the RBD protein. Si corresponds to the underlying electrode substrate

The SPCE before and after the addition of the SARS-CoV-2 Spike RBD protein was characterized using SEM to verify successful immobilization on the electrode surface. Figure 9 shows the results of the SEM and EDX mapping of SPCE/CeO_2_-Au/cysteamine/RBD.

The results show that immobilization was successful, with an object covering the electrode surface. This is supported by the EDX mapping results, which show that S and N are constituent elements of cysteamine, as are amino acids from the RBD. Ce, O, and Si are the constituent elements of CeO_2_-Au. Na is an element of the RBD solvent used, namely the PBS solution.

### Electrochemical characterization

**Figure 10.**
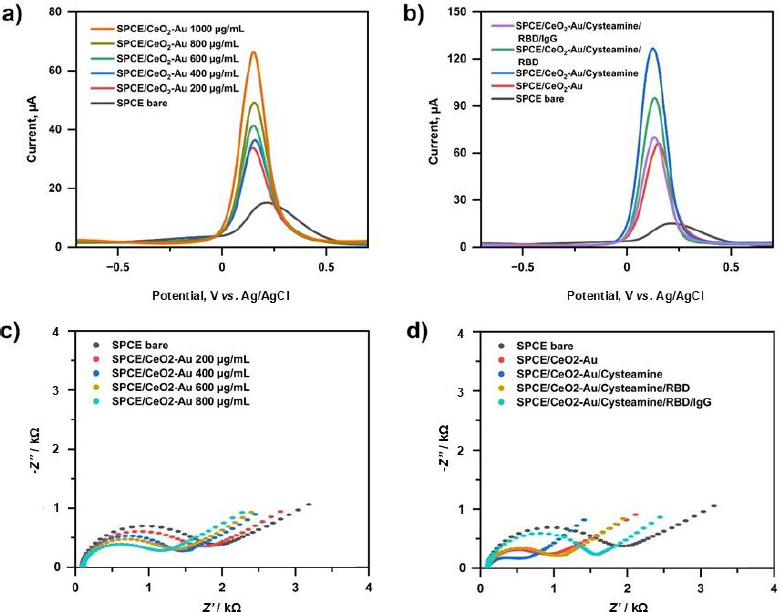
Electrochemical characterization of modified SPCE electrodes using 10 mM [Fe(CN)_6_]^3-^/^4-^ in 0.1 M KCl. (a) Differential pulse voltammograms (DPV) of SPCE modified with varying concentrations of CeO_2_-Au nanocomposites, showing progressive enhancement of peak current. (b) DPV responses of SPCE at each stage of surface modification. (c) Nyquist plots of SPCE modified with different CeO_2_-Au concentrations, illustrating changes in charge transfer resistance (*R*_ct_). (d) Nyquist plots corresponding to each immobilization step, recorded over a frequency range of 0.1 to 10^6^ Hz at an applied potential of 0.01 V. Extracted *R*_ct_ values were obtained using the Randles equivalent circuit model

The SPCE before and after modification with the CeO_2_-Au nanocomposites was electrochemically characterized by DPV and EIS (Figure 10).

Based on the voltammogram in Figure 10a, the modified electrode shows a higher current than the unmodified electrode. This indicated that the SPCE modification process was successful. Modification of CeO_2_-Au nanocomposites increases conductivity, thereby improving electron transfer between the electrode and the analyte. On the other hand, the EIS characterization results in Figure 10c show a decrease in the impedance. This result was inversely proportional to the results obtained from the current measurements. This is because, according to Ohm's law, resistance and current are inversely proportional: *V* = *IR.*

The SPCE surface was blocked with 1 % (w/v) BSA to prevent nonspecific binding to unoccupied sites not covered by RBD. After each immobilization step, the SPCE was washed with PBS pH 7.4 to remove loosely bound species from the surface. This washing step is essential to minimize measurement errors in the immunosensor response, as unattached species could produce lower-than-expected signals and interfere with the analytical process. During immunosensor testing, IgG binds specifically to the RBD immobilized on the SPCE surface.

To prove the potential of the synthesized CeO_2_-Au nanocomposites in SPCE modification, we also characterized DPV against bare SPCE and modified SPCE with CeO_2_, AuNP and CeO_2_-Au in RBD immobilization (Figure S1, Supplementary material). Based on these results, it can be concluded that the CeO_2_-Au potential not only enhances the detection signal but also increases the electrode surface's adsorption capacity during RBD protein immobilization. The concentration of the CeO_2_-Au nanocomposites was determined by modifying the SPCE at a concentration variation of 200 to 1000 μg mL^-1^.

As shown in Figure 10, the voltammetric results show that the bare SPCE exhibited a low peak current of approximately 14 μA, which increased significantly after modification with CeO_2_-Au nanocomposites. The peak current rose progressively from ~35 μA (200 μg mL^-1^) to ~64 μA (1000 μg mL^-1^), indicating enhanced electron transfer and increased electroactive surface area. After cysteamine modification, the current further increased to ~124 μA, while subsequent immobilization of RBD and IgG decreased the current to ~94 and ~72 μA, respectively, due to the insulating nature of the protein layers.

Consistently, EIS analysis showed that the bare SPCE had a high charge transfer resistance (*R*_ct_) of ~1.6 kΩ, which decreased to ~1 kΩ at higher CeO_2_-Au loading, confirming improved conductivity. Following cysteamine modification, the Rct further decreased to ~0.4 kΩ, indicating enhanced electron transfer due to the presence of conductive amine-functionalized linker molecules. After RBD and IgG immobilization, the *R*_ct_ increased to ~0.8 and ~1.4 kΩ, respectively, reflecting hindered electron transfer upon antigen-antibody binding. These complementary changes in current and *R*_ct_ confirm successful stepwise fabrication of the immunosensor.

The SARS-CoV-2 Spike RBD protein is immobilized on the SPCE surface through electrostatic interactions between the positively charged group of cysteamine and the negatively charged groups on the protein. The NH_2_ group in the system facilitates proper orientation of RBD immobilization, with the protein's negatively charged groups (including the C-terminus) positioned toward the surface, allowing the protein epitope to remain accessible for binding to the antibody paratope. Although the experiments were conducted under pH conditions (PBS pH 7.4) below the isoelectric point of the protein (pI = 8.55), the negatively charged groups on the protein are not fully protonated because the H^+^ concentration is relatively low; therefore, negatively charged groups remain present on the protein surface. In addition, it has been reported that over a wide pH range, the lower region of the RBD tends to be negatively charged, while the upper region remains positively charged, facilitating binding to antibody paratopes [59].

### Optimum conditions for immunosensor assembly parameters

Three factors, namely RBD S concentration (*X*_1_), RBD S incubation time (*X*_2_) and IgG incubation time (*X*_3_) were selected as factors to be optimized in the experiment using the Box-Behnken experimental design. Each factor was designed through 3 different levels. The experiment was carried out 15 times, as shown in Table S1. The response (current signal) was analysed in Minitab 19 to fit a quadratic regression model.

From each experiment, the following regression equation was obtained:



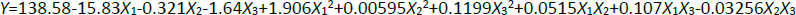

(7)


Although the regression model generated predictive coefficients for linear, quadratic, and interaction terms, ANOVA analysis showed that the linear terms were not statistically significant at the 95 % confidence level (*p* >0.05). The *p*-values for RBD S concentration, RBD S incubation time, and IgG incubation time were 0.506, 0.458 and 0.704, respectively. These results indicate that, within the investigated experimental range, none of the individual linear factors had a statistically significant effect on the response.

Nevertheless, response surface methodology (RSM) was used to explore the predicted response behaviour within the design space. The model predicted optimum conditions at an RBD S concentration of 3.25 μg mL^-1^, RBD S incubation time of 43 minutes, and IgG incubation time of 11 minutes (Figure S2). These conditions correspond to the maximum predicted response within the tested parameter domain, rather than statistically significant factor effects.

**Figure 11.**
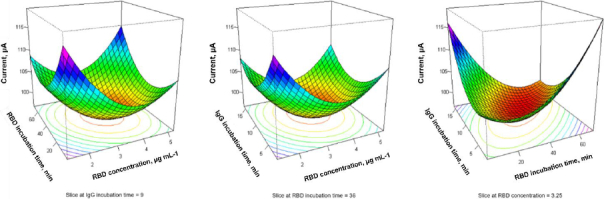
Surface plots derived from the Box-Behnken design illustrating the predicted response of the immunosensor as a function of RBD concentration, RBD incubation time and IgG incubation time

The surface plots (Figure 11) illustrate the distribution of the response across the studied parameter space. While no statistically significant individual effects were observed, the response surface analysis provides a visualization of system behaviour and supports the selection of practical operating conditions within the experimental region. Therefore, the Box-Behnken design in this study serves primarily as a systematic multivariate screening and prediction tool rather than a definitive statistical confirmation of significant factor effects.

### Calibration curves, detection limits and quantification limits

A calibration curve was constructed to evaluate the analytical performance of the developed immunosensor, including linearity, sensitivity, limit of detection (LOD), and limit of quantification (LOQ). The immunosensor was tested at different IgG concentrations of 0, 0.01, 0.1, 1, 10, 100, and 1000 ng mL^-1^. Each concentration was measured in triplicate (*n* = 3), and the results are expressed as mean ± standard deviation.

**Figure 12.**
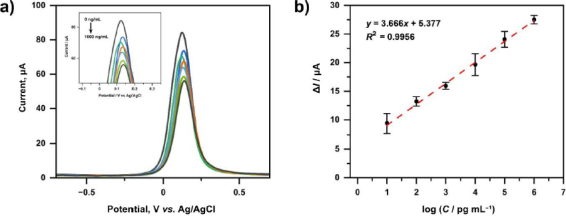
(a) Differential pulse voltammogram for variations in IgG concentration (0-1000 ng/ml) and (b) Calibration curve of IgG immunosensors with concentration variations measured by redox system [Fe(CN)_6_]^3+/4+^ 10 mM in KCl 0.1 M

The electrochemical response was recorded using differential pulse voltammetry (DPV), as shown in Figure 12a. The peak current decreased progressively with increasing IgG concentration. This behaviour can be attributed to the formation of an insulating immunocomplex layer. Since IgG is a relatively large biomolecule, it’s binding to Spike RBD increases surface coverage on the SPCE, thereby hindering electron transfer between the redox probe and the electrode surface.

The calibration curve (Figure 12b) was constructed by plotting the peak current versus the logarithm of IgG concentration. A linear relationship was observed within the tested concentration range, with the regression equation *y* = 3.666*x* + 5.377 and a correlation coefficient of *R*^2^ = 0.9956, indicating excellent linearity.

The LOD and LOQ were calculated from the standard deviation of the y-intercept (*σ*) and the slope (*S*) of the calibration curve, using the equations: LOD = 3*σ*/*S*, and LOQ = 10*σ*/*S*. The calculated LOD and LOQ were 2.475 and 15.59 pg mL^-1^, respectively. The LOQ is slightly higher than the lowest concentration in the linear range (10 pg mL^-1^), which is expected, as the LOQ represents the minimum concentration that can be quantified with acceptable precision and accuracy. The use of the regression-based approach accounts for variability in the calibration model and ensures statistically reliable quantification. The precision of the immunosensor was evaluated in terms of repeatability, with RSD values below 2.85%, demonstrating good analytical reproducibility.

### Immunosensor selectivity test

**Figure 13.**
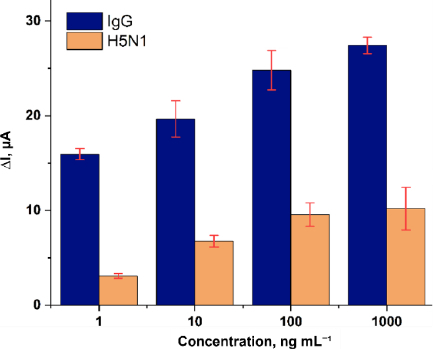
Selectivity test results of the Immunosensor: Comparison of Δ*I* for IgG and H5N1 at various concentrations

The selectivity of the immunosensor was evaluated by comparing its response toward the target analyte (IgG) and a non-target protein (H5N1) over a concentration range of 1 to 1000 ng mL^-1^. As shown in Figure 13, the Δ*I* response for IgG is consistently higher than that for H5N1 at all tested concentrations.

At 1 ng mL^-1^, IgG produced a Δ*I* of 15.945 μA, while H5N1 generated 3.077 μA, corresponding to approximately a 5.2-fold higher response for IgG. At 1000 ng mL^-1^, the response toward IgG (27.423 μA) remained approximately 2.7 times higher than that of H5N1 (10.201 μA).

The consistently higher signal toward IgG across the entire concentration range indicates preferential binding of the immobilized antibody toward its specific antigen. Although only one potential interfering protein was evaluated, the significant signal discrimination suggests that the fabricated immunosensor exhibits acceptable selectivity under the tested conditions.

### Immunosensor stability test

To evaluate the practical storage requirements of the developed immunosensor, a factorial design approach was applied to simulate realistic laboratory and handling conditions. Four storage-related factors were investigated: stabilizing solution (Factor A), packaging condition (Factor B), temperature (Factor C), and humidity (Factor D). These factors were selected because they represent common conditions encountered during laboratory storage, transportation, and potential field deployment.

Factor A examined the addition of stabilizing agents (NaN_3_, Na_2_EDTA, BSA in PBS), which are commonly used in protein preservation. Factor B evaluated open versus closed packaging to simulate exposure to dust and ambient light during handling. Factor C compared room temperature (RT = 25±2 °C) and refrigeration (4 to 5 °C), reflecting typical laboratory and clinical storage environments. Factor D assessed humidity control using silica gel, considering that ambient laboratory humidity ranged from 64 to 76 %, while refrigeration humidity ranged from 38 to 52 %.

Statistical analysis using R software demonstrated that only the stabilizing solution had a statistically significant effect on current response decrease (*p* = 0.0185, *p* < 0.05), while packaging (*p* = 0.4153), temperature (*p* = 0.2304) and humidity (*p* = 0.2542) showed no significant individual effects within the one-week study. However, based on the main effect plot, the optimal practical storage condition corresponded to: no stabilizing solution, closed packaging, low temperature (4-5 °C), and low humidity (<20 %). This condition resulted in the smallest current reduction and was therefore selected for long-term shelf-life studies.

Temperature can affect the stability of biomolecules because high temperatures can denature proteins, causing them to lose their ability to bind to specific targets [60]. In addition, proteins stored at room temperature and exposed to external particles can be degraded and/or rendered inactive by microbial growth. Therefore, proteins stored at 4 °C under closed conditions can slow microbial growth, making them more stable.

Proteins are also sensitive to light exposure, leading to protein oxidation [61]. Aromatic amino acids, tryptophan, phenylalanine, tyrosine and cysteine, containing sulphur, can absorb UV light in the range of 250 to 300 nm and are excited to an electronic state, producing amino acid radicals. The released energy can be absorbed by oxygen to produce reactive oxygen species (ROS), which can oxidize amino acids [62]. Fluorescent lamps are the most widely used indoor light source in laboratories, offices and manufacturing areas. Commercial fluorescent lamps emit visible light and small amounts of UV light [63]. Therefore, exposure to light can affect protein stability, so proteins stored in a closed state are more stable.

On the other hand, humidity is related to the amount of water vapor in the air around the immunosensor. High humidity increases the amount of water available to dissolve proteins and encourages deactivation through unfolding [64]. So that proteins stored at high air humidity have decreased stability.

**Figure 14.**
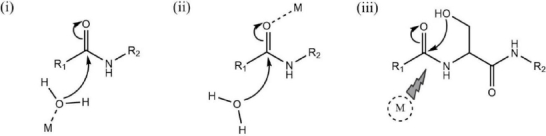
General mechanisms of hydrolysis of peptide bonds by metal ions: (i) activation of water molecules by metal ions; (ii) polarization of the carbonyl group by interaction of metal ions with amide oxygen; and (iii) acyl rearrangements containing hydroxyl or carboxyl groups in side chain residues. Adapted with modification from ref [67]

To study the effect of stabilizer type, Figure S5 shows voltammogram results from eight sets of factorial design experiments, separated by the addition of stabilizer solution to the current response. The addition of a stabilizing solution causes a greater decrease in current than without its addition. This is because EDTA is a strong chelating agent that can dissolve/release Ce(III) on the surface of CeO_2_ [65]. Ce^3+^ ions can hydrolyse peptide bonds in proteins, as shown in Figure 14.

Metal ions can activate water molecules (i) so that carbonyl oxygen binds to peptide bonds (ii), then acyl shifts/rearrangements occur (protein self-division) and (iii) Ce^3+^ ions are found to hydrolyse peptide bonds [66].

From a practical perspective, storage at 4-5 °C in closed packaging is highly feasible in clinical laboratories and diagnostic centres, where refrigeration is routinely available. Additionally, the use of silica gel for humidity control is inexpensive and easily implemented in commercial packaging formats (*e.g.* sealed foil pouches).

**Figure 15.**
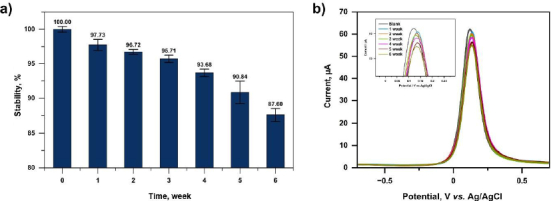
Results of the immunosensor shelf life stability study for six weeks (a) histogram (b) differential pulse voltammogram with redox system [Fe(CN)_6_]^3+/4+^ 10 mM in KCl 0.1 M

Long-term stability testing was conducted over six weeks under the optimized storage conditions. The immunosensor response was evaluated weekly using anti-SARS-CoV-2 IgG (1 ng mL^-1^). The signal decrease over six weeks was 2.27, 3.28, 4.29, 6.32, 9.16 and 12.4 %, respectively (Figure 15). The immunosensor maintained more than 90 % of its initial signal for up to five weeks, meeting the stability criterion reported by Liu *et al*. [66], which considers sensors unstable when signal loss exceeds 10 %.

These results indicate that the developed immunosensor is suitable for short- to medium-term storage (up to 5 weeks) under refrigeration without chemical stabilizers. This storage profile is compatible with centralized production and refrigerated distribution to clinical laboratories. However, for applications requiring extended room-temperature storage (*e.g.* point-of-care settings in resource-limited environments), further stabilization strategies may be necessary.

### Human serum sample analysis

**Figure 16.**
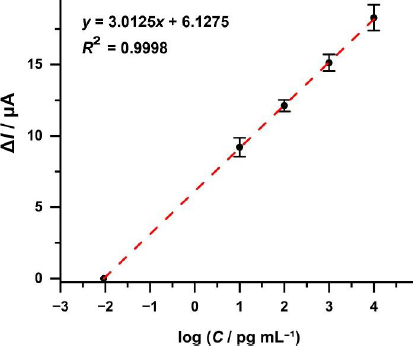
Standard curve of the addition of serum samples with standard concentration variation to the decreasing current response.

Immunosensor testing on human serum samples was conducted to evaluate the validity of the test. The testing was performed on patient serum using the standard addition method, where standard solutions with varying concentrations were added to the diluted serum samples. The resulting standard addition curve is shown in Figure 16, with the line equation *y* = 3.0125*x* + 6.1275 and an *R*^2^ value of 0.9998.

Using the developed biosensor method, the sample concentration was determined to be 0.7111±0.004 pg mL^-1^ or 5,640.9±31.4 AU mL^-1^. These results were compared with those obtained using the CMIA (chemiluminescent microparticle immunoassay) method at the Prodia Clinic, which yielded a concentration of 0.6575 pg mL^-1^ or 5,215.9 AU mL^-1^. Clinical guidelines state that individuals are classified as reactive and eligible to donate plasma if their IgG levels are ≥50 AU mL^-1^. In this case, the patient, a COVID-19 survivor who has been vaccinated, met the eligibility criteria. The comparison between the two methods showed consistent results, with the CMIA method demonstrating slightly higher sensitivity than the developed biosensor, with a difference of approximately 8.15 %.

Immunosensors were then used to determine the recovery (R / %) against anti-SARS-CoV-2 IgG by adding a standard amount to serum samples. The results obtained are shown in Table 3. *R* IgG anti-SARS-CoV-2 in human serum samples using the immunosensors developed were in the range of 97.3 to 108.56 %. This figure is acceptable, where the *R* acceptance at analyte concentrations of 1 to 10 ng mL^-1^ is 40 to 115 % [68].

**Table 3.** Recovery of anti-SARS-CoV-2 IgG in human serum samples with different concentrations

Real concentration of IgG anti-SARS-CoV-2], ng mL^-1^	Measured concentration of IgG anti-SARS-CoV-2, ng mL^-1^	*R* / %
0.01	0.010547	105.47
0.10	0.097671	97.67
1.00	0.973000	97.30
10.00	10.856000	108.56

Table 4 compares various biosensor platforms based on the transducer used, LoD and LoQ, long-term stability, recovery rate, sample type, and related research references. Based on the data in the table, the GCE/Au-based biosensor showed the highest sensitivity, with a LoD of 0.01 ag mL^-1^, while SPCE/NiONP also exhibited excellent sensitivity, with a LoD of 0.3 fg mL^-1^. In this study, the SPCE/CeO_2_-Au-based biosensor had a LoD of 2.475 pg mL^-1^ and a LoQ of 15.59 pg mL^-1^. These results indicate that the developed biosensor has competitive sensitivity for detecting Anti-SARS-CoV-2 IgG.

**Table 4.** Comparison of different biosensor platforms for SARS-CoV-2 antibody detection

Transducer	LoD	LoQ	Long-term stability	Linear range, ng mL^-1^	Assay time, min	Sample	Ref.
SPCE/NiONP	0.3 fg mL^-1^	NR*	14 days	10^-6^ to 10^3^	20	NR*	[38]
GCE/Au	0.01 ag mL^-1^	NR*	30 days	10^-10^ to 10^-6^	30	Saliva	[68]
SPTE	10.1 ng mL^-1^	NR*	24 weeks	10.1 to 6.0×10^4^	13	Serum	[69]
SPCE/PANI	3.9 ng mL^-1^	NR*	NR*	10 to 10^4^	NR*	Serum	[70]
SPCE/GQD	2.028 ng mL^-1^	NR*	NR*	0.5 to 10^2^	15	Serum	[71]
SPCE/PANI	8.00 ± 0.20 nM	23.93 ± 0.60 nM	NR*	0 to 10^3^	NR*	Serum	[72]
SPCE/HAG**	0.056 pg mL^-1^	0.17 pg mL^-1^	7 weeks	10^-3^ to 10^2^	10	Serum	[73]
SPCE/CeO_2_-Au	2.475 pg mL^-1^	15.59 pg mL^-1^	42 days	10^-2^ to 10^3^	11	Serum	This work

*Not reported

**hydroxyapatite-gold

Long-term stability is an important parameter for practical applications. GCE/Au-based biosensors are stable for up to 30 days, while SPTE is stable for up to 24 weeks. In this study, the SPCE/CeO_2_-Au biosensor showed quite good stability for 42 days, which is higher than that of several other SPCE-based platforms that did not report their stability data. The recovery rate describes the accuracy of the biosensor in measurement. The recovery of the biosensor in this study ranged from 97.3 to 108.56 %, which is equivalent to, or even better than, other platforms such as GCE/Au (96.97 to 101.99 %) and SPCE/GQD (99.2±7.8 %). Regarding sample type, most studies use serum as the analytical matrix, including this study. However, some platforms, such as GCE/Au, also use saliva as an alternative sample. The main advantage of this study is the use of CeO_2_-Au nanocomposite, which has never been reported before, to detect Anti-SARS-CoV-2 IgG. The use of CeO_2_-Au provides high sensitivity, adequate stability, and good accuracy, making this biosensor a potential alternative for serological applications, supporting the evaluation of vaccine effectiveness and the detection of SARS-CoV-2 antibodies.

Although some previously reported platforms exhibit lower limits of detection, these systems often involve multi-step fabrication processes or limited validation in real samples. In contrast, the proposed SPCE/CeO_2_-Au platform demonstrates a balanced performance profile, combining competitive sensitivity with acceptable stability and practical assay time, making it suitable for routine analytical applications.

## Conclusion

The incorporation of CeO_2_- Au nanocomposites into electrode modification enhanced the signal response and facilitated biomolecular immobilization. The developed SPCE/CeO_2_-Au-based electrochemical immunosensor demonstrated high sensitivity for anti-SARS-CoV-2 IgG detection over a linear range of 0.01 to 1000 ng mL^-1^, with limits of detection (LOD) and quantification (LOQ) of 2.475 and 15.59 pg mL^-1^, respectively. The immunosensor retained 90.84 % of its initial response after five weeks of storage, indicating acceptable storage stability. Recovery studies in spiked human serum samples showed satisfactory results (97.3 to 108.56 %), suggesting good analytical accuracy. These findings indicate that the developed immunosensor has promising potential for serum sample analysis; however, further validation using a larger number of clinical samples is required to confirm its practical applicability.

## Supplementary material

Additional data are available at https://pub.iapchem.org/ojs/index.php/admet/article/view/3182, or from the corresponding author on request.



## List of abbreviations

APTES: (3-aminopropyl)triethoxysilane
AU: Arbitrary unit
Au: Gold
AuNP: Gold nanoparticle
BSA: Bovine serum albumin
CeO_2_: Cerium oxide
CeO_2_-Au: Cerium oxide-gold nanocomposite
CLIA: Chemiluminescent immunoassay
CMIA: Chemiluminescent microparticle immunoassay
COVID-19: Coronavirus disease 2019
DPV: Differential pulse voltammetry
EDX: Energy-dispersive X-ray spectroscopy
EIS: Electrochemical impedance spectroscopy
ELISA: Enzyme-linked immunosorbent assay
FTIR: Fourier transform infrared spectroscopy
GCE: Glassy carbon electrode
IgA: Immunoglobulin A
IgG: Immunoglobulin G
K_3_[Fe(CN)_6_]: Potassium ferricyanide
IgM: Immunoglobulin M
KCl: Potassium chloride
LFA: Lateral flow assay
LoD: Limit of detection
LoQ: Limit of quantification
PBS: Phosphate-buffered saline
PRNT: Plaque reduction neutralization test
RBD: Receptor-binding domain
RSD: Relative standard deviation
RT: Room temperature
SARS-CoV-2: Severe acute respiratory syndrome coronavirus 2
SEM: Scanning electron microscopy
SPCE: Screen-printed carbon electrode
SPR: Surface plasmon resonance
TEM: Transmission electron microscopy
UV-Vis: Ultraviolet-visible spectrophotometry
WHO: World Health Organization
XRD: X-ray diffraction
