# Supplementary material for: High-sensitivity electrochemical immunosensor for anti-SARS-CoV-2 IgG detection using screen-printed carbon/cerium oxide-gold electrode
Source: ADMET DMPK. 2026 Apr 10;14:3182. doi: 10.5599/admet.3182 (PMC13147514; doi:10.5599/admet.3182)
Supplement: Supplementary file 1 [file ADMET-14-3182-S1.pdf]

ADMET & DMPK **14** (2026) S3182

Open Access : ISSN : 1848-7718
http://www.pub.iapchem.org/ojs/index.php/admet

Supplementary material to

High-sensitivity electrochemical immunosensor for anti-SARS-CoV-2 IgG detection using screen-printed carbon/cerium oxide-gold electrode

Melania Janisha Devi[1], Ratu Shifa Syafira[1], Shabarni Gaffar[1], Irkham Irkham[1], Yasuaki Einaga[2] and Yeni Wahyuni Hartati[1,*]

[1]*Department of Chemistry, Faculty of Mathematics and Natural Sciences, Padjadjaran University, Jl. Raya Bandung-Sumedang Km 21, Jatinangor, Sumedang, West Java 45363, Indonesia*
[2]*Department of Chemistry, Keio University, 3-14-1 Hiyoshi, Yokohama, 223-8522, Japan*

ADMET & DMPK **14** (2026) 3182; https://doi.org/10.5599/admet.3182

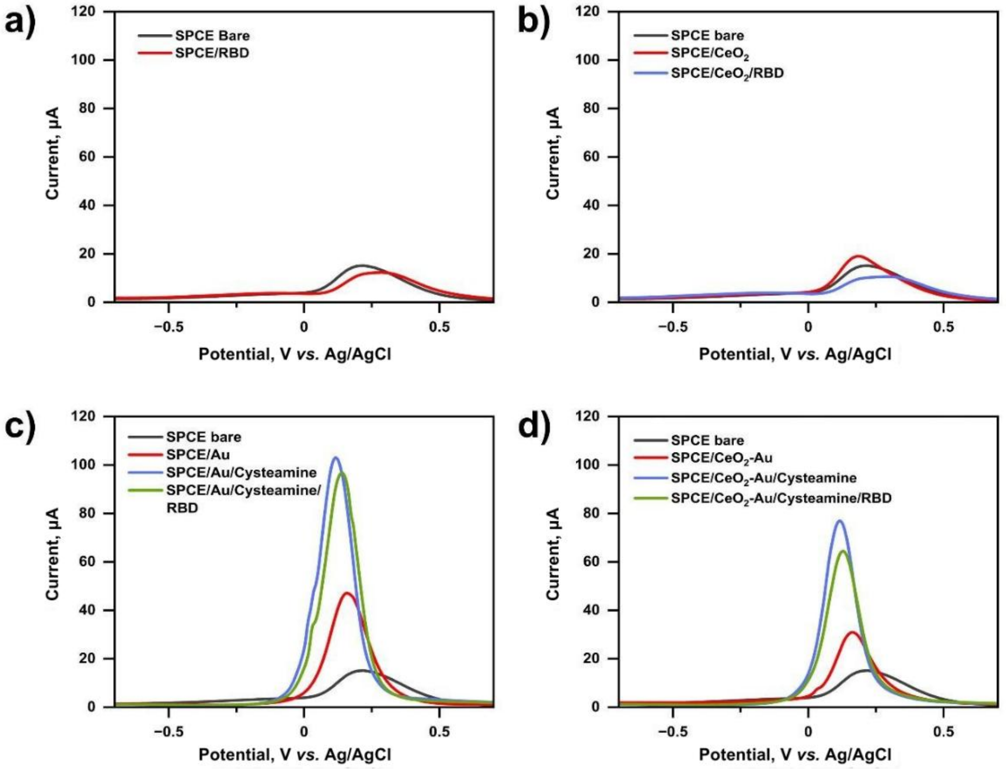

Figure S1. Differential pulse voltammograms of each modification stage for (a) unmodified SPCE (SPCE bare), and SPCEs modified with (b) CeO_2, (c) AuNP and (d) CeO_2-Au with the same suspension/colloidal concentration using $[Fe(CN)_6]^{3+/4+}$ 10 mM redox system in 0.1 M KCl

Table S1. Data on the peak height of potassium ferricyanide on variations in IgG concentrations

IgG concentration, ng mL^{-1}	log (C_{IgG} / pg mL^{-1})	ΔI ± SD / μA
0.01	1	9.423 ± 1.676
0.1	2	13.219 ± 0.816
1	3	15.945 ± 0.587
10	4	19.659 ± 1.917
100	5	24.066 ± 1.372
1000	6	27.508 ± 0.732

Linier regression :

$y = 3.666x + 5.377$

$$\text{Standard error} = \sqrt{\frac{\sum \left(y\text{-}y_{\text{predict}}\right)^2}{N\text{-}2}} = 0.469$$

$$\sigma \text{ intercept} = \text{SE}\sqrt{\frac{\sum Xi^2}{N\sum \left(x\text{-}\underline{x}\right)^2}} = 0.437$$

$$\text{LoD} = \frac{3\sigma}{\text{Slope}} = 2.475 \text{ pg mL}^{-1}$$

$$\text{LoQ} = \frac{10\sigma}{\text{Slope}} = 15.588 \text{ pg mL}^{-1}$$

Table S2. Precision data

IgG concentration, ng mL^{-1}	ΔI / µA	Average ΔI ± SD / µA
1.0	11.910	15.129 ± 1.915
	15.267	
	16.286	
	16.283	
	17.094	
	13.941	

$$\text{CV*} = \frac{SD}{\underline{I}}\ 100 = 2.855\ \%$$

Precision = 100 - CV = 97.145 %

*In probability theory and statistics, the coefficient of variation (CV), also known as relative standard deviation (RSD)

Accuracy

$X_{\text{observed}} = 2.66$

$X_{\text{true}} = 3$

$$\text{Error} = \left|\frac{X_{\text{observed}} - X_{\text{true}}}{X_{\text{true}}}\right| 100 = 11.326\ \%$$

Accuracy = 100 - Error = 88.674 %

Table S3. Results of fractional design with eight experiments under different immunosensor storage conditions

**No**	Addition of stabilizing solution	Packaging	Temperature, °C	Humidity	ΔI ± SD / µA
1	Yes	Open	4 to 5	Low	41.648 ± 4.367
2	No	Closed	4 to 5	Low	1.373 ± 4.344
3	No	Closed	RT	Normal	25.156 ± 1.441
4	No	Open	4 to 5	Normal	12.299 ± 1.597
5	No	Open	RT	Low	26.465 ± 0.480
6	Yes	Closed	RT	Normal	48.603 ± 0.304
7	Yes	Closed	RT	Low	35.580 ± 5.645
8	Yes	Open	RT	Normal	53.250 ± 0.669

CC BY

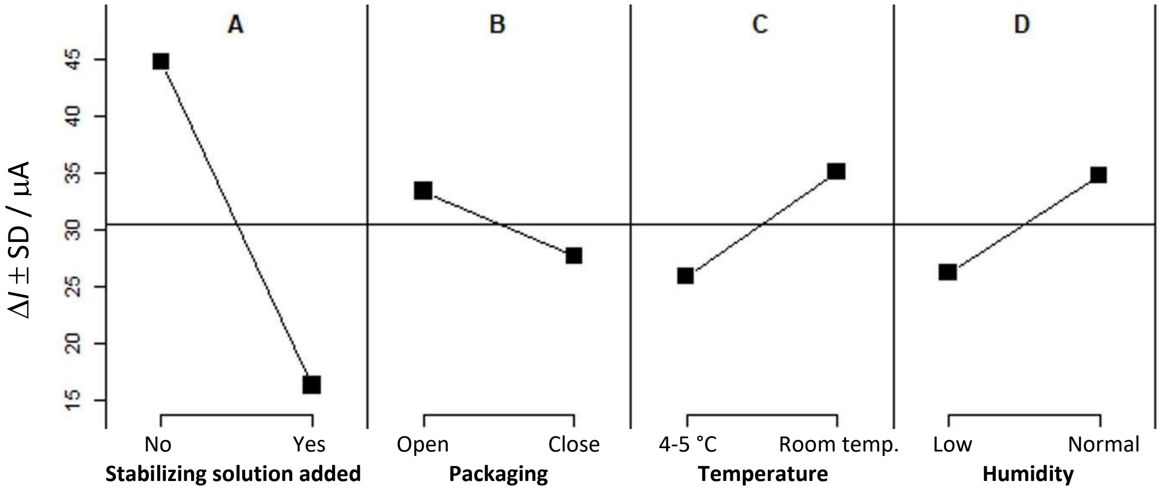

Figure S2. Main effect plot based on immunosensor storage factors for one week against current change response. (Factors: A = addition of stabilizing solution, B = packaging, C = temperature and D = humidity).

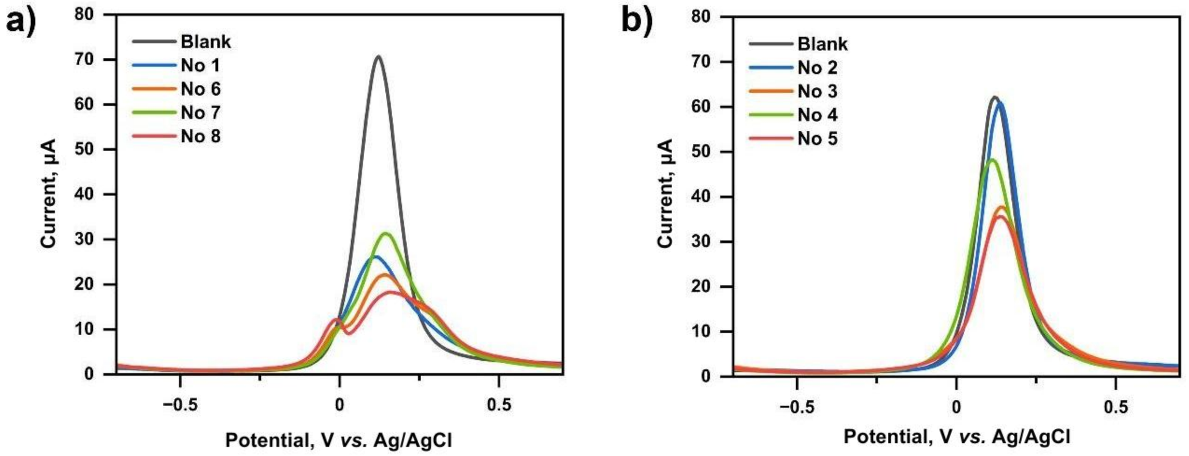

Figure S3. Differential pulse voltammogram with differences in the addition of stabilizer solutions in eight fractional design experiments where (a) using stabilizer solutions (consisting of NaN_3, Na_2EDTA, BSA and PBS) and (b) not using stabilizing solutions using redox systems $[Fe(CN)_6]^{3+/4+}$ 10 mM in KCl 0.1 M
